# Ascending colon stenosis caused by repeated diverticulitis that clinically mimicked advanced colon cancer: A case report

**DOI:** 10.1016/j.ijscr.2022.107184

**Published:** 2022-05-12

**Authors:** Shogo Yoshida, Kazuhiro Hiyama, Izumi Kirino, Yasuo Fukui, Hideo Terashima

**Affiliations:** a Kochi Medical School (KMS), Kochi University, Kochi, Japan; b Department of Surgery, Atago Hospital, Kochi, Japan

**Keywords:** Diverticulitis, Right-sided large bowel obstruction, Interval surgery

## Abstract

**Introduction:**

We experienced a rare case of right-sided large bowl obstruction (LBO) of the colon caused by chronic diverticulitis, which was challenging to diagnose.

**Presentation of case:**

A young male was admitted to our department with a fever, diarrhea, and right-sided lateral abdominal pain for several days. CT showed a thickened ascending colon wall with stenosis and adjacent retroperitoneal inflammation without marked diverticula. The next day, he developed severe abdominal pain, and perforation was suspected. We chose the “interval definitive surgery”; at that time, intestinal decompression and laparoscopic drainage. Colonoscopy showed an edematous membrane, but no cancerous lesions or diverticula. Hemi-colectomy was performed after 10 days' nutritional therapy. No postoperative complication occurred. The histopathology showed that the pathogenesis was chronic diverticulitis.

**Discussion:**

There have been few reported cases of right-sided LBO caused by diverticulitis, but it is important to be aware that benign disease, such as chronic diverticulitis, can cause LBO. Initial conservative therapy and nutritional therapy produced a correct diagnosis and good outcomes.

**Conclusion:**

Performing “interval surgery” allowed us to make an accurate diagnosis and may help to prevent surgical complications in rare cases of right-sided LBO due to diverticulitis.

## Highlights

• Most cases of large bowel obstruction (LBO) are caused by cancer.
• Right-sided LBO of the colon due to chronic diverticulitis is rare.
• Definitive interval surgery leads to better outcomes and reduces perioperative complications.

## 1 Introduction

Diverticular disease, including diverticulitis, is one of the major causes of hospitalization in Japan and the United States [1]. The prevalence of diverticulosis, a potential cause of diverticulitis, is increasing among Japanese people [1]. In diverticulosis, the affected site varies depending on genetic and lifestyle factors [1], [2], [3]. Most Caucasians tend to develop diverticulosis on the left side. On the other hand, Asian people tend to develop it on the right side [1], [2], [3]. The incidence of diverticulitis among people with diverticulosis was estimated to be 6 per 1000 patient-years, and the risk of diverticulitis decreases with age [4], contrary to the risk of diverticulosis, which increases with age [2].

Most cases of large bowl obstruction (LBO) are caused by left-sided colon cancer [5], almost all of which is advanced, whereas it occurs infrequently on the right side of the colon because the contents of this section of the colon are watery and easily passed. Diverticulitis can sometimes cause LBO; i.e., it accounts for 4–10% of cases of LBO [5], but most previously reported cases of LBO associated with diverticulitis involved the left side of the colon, especially the sigmoid colon.

## 2 Presentation of case [6]

**Fig. 1:**
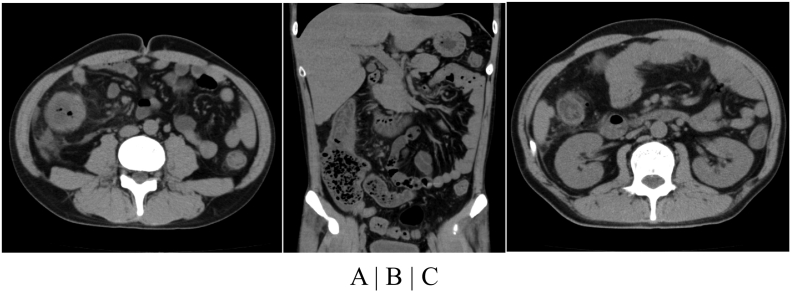
Plain CT findings. (A) A plain CT scan obtained on admission showed a thickened ascending colon wall with stenosis, a ballooning cecum, and high-density lesions in the retroperitoneum. (B) Oral intestinal distention was observed. (C) A plain CT scan obtained the day after admission showed small air bubbles besides the ascending colon without fluid collection.

A 43-year-old Japanese male presented to a nearby clinic with a high fever and right-sided lateral abdominal pain and was referred to our hospital for further investigation. He stated that he had not experienced any previous similar episodes and did not have an abnormal diet. He did not have any medical comorbidities and was not taking any medication. He smoked one pack of cigarettes per day and occasionally drank alcohol. A physical examination showed slight abdominal distention and right iliac hardness. His bowel sounds were diminished. A plain computed tomography (CT) scan demonstrated marked wall thickening in the ascending colon with intestinal distention and adjacent retroperitoneal inflammation, but did not show marked diverticulosis (Fig. 1A and B). He showed signs of inflammation (C-reactive protein level: 8.7 mg/dL, white blood cell count: 12,840/μL). Enteritis was selected as the initial differential diagnosis, but the patient did not exhibit hematochezia, which is mainly seen in infectious colitis. Although the CT images did not show the typical findings of colon cancer, we considered colon cancer as another possible differential diagnosis. Incidentally, his carcinoembryonic antigen (CEA) level was slightly elevated (9.5 ng/mL). Since his abdominal pain was not severe, we decided to admit him and conduct further investigations.

### 2.1 Evaluations and management

We had planned to perform colonoscopy after several days' fasting. However, the patient's abdominal pain worsened the next morning. A plain CT scan showed small air bubbles besides the ascending colon without fluid collection, which suggested micro-perforation of the distended intestine (Fig. 1C). At this point, since his peritonitis was locally limited, and we had not made a final diagnosis, we decided to attempt to perform exploratory laparoscopy and irrigation without emergent colectomy.

**Fig. 2:**
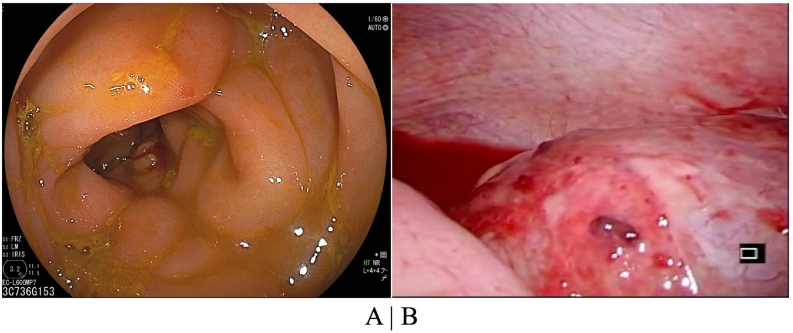
Findings obtained during the first surgery. (A) Findings of colonoscopy: An edematous membrane without any obvious cancer lesions was seen. (B) Findings of laparoscopy: Hemorrhagic ascites and marked mesenteric disease were observed, but no rupturing or cancer lesions were seen.

First, a long transnasal tube was inserted for oral intestinal decompression. Then, preoperative colonoscopy was conducted, which showed stenosis of the ascending colon due to external compression. However, the colonoscope easily passed through the stenotic region. In addition, an edematous membrane was observed, but no obvious cancerous lesions or diverticula were noted (Fig. 2A). Thirdly, exploratory laparoscopy revealed hemorrhagic ascites, marked mesenteric disease, and extremely stiff fibrous tissue around the stenotic region, but no obvious rupturing or cancer lesions (Fig. 2B). These findings suggested that the bowel stenosis was ascribable to compression triggered by a condition such as panniculitis. As planned, we performed irrigation and placed a drainage tube without performing colorectomy.

**Fig. 3:**
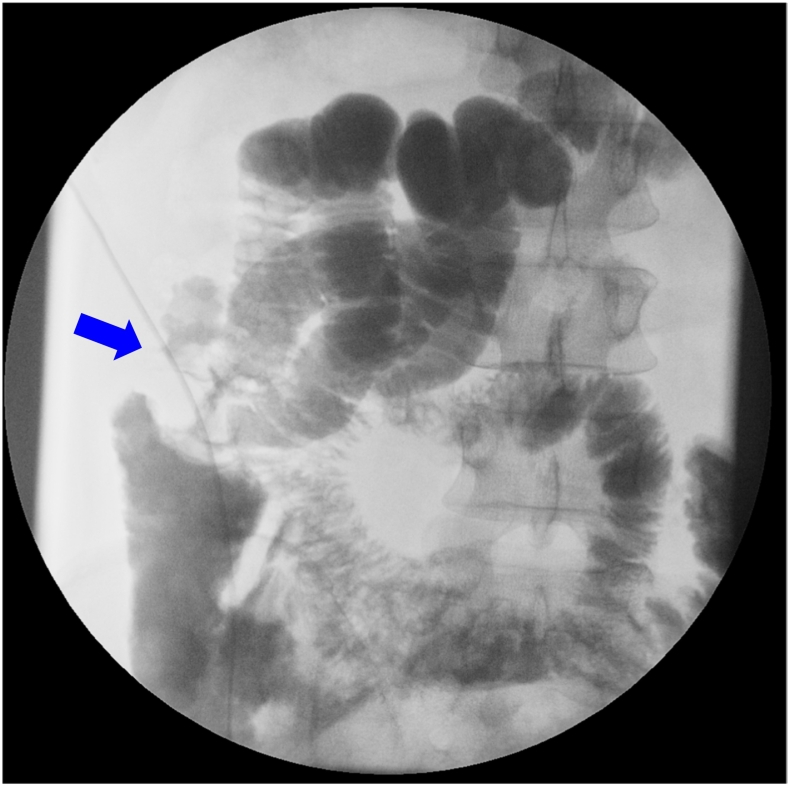
A radiographic contrast enema performed after the first surgery. A long radiopaque transnasal tube was inserted. Stenosis still remained (blue arrow), but fluid easily passed through the lesion. (For interpretation of the references to colour in this figure legend, the reader is referred to the web version of this article.)

The patient's peritonitis had completely resolved by postoperative day 4. A radiographic contrast enema performed through a long nasal tube showed the passage of fluid through the stenotic region, but severe stenosis was still observed (Fig. 3). The pathogenesis of the condition was unclear, but it was considered that solid food intake would probably cause the obstruction to recur, although the stenosis may be improved if panniculitis had caused it and had subsequently been ameliorated. The patient wanted to resume food intake and undergo resection due to his expectation that the condition would recur and its pathogenesis was obscure. Therefore, laparoscopic right hemicolectomy was carried out after 10 days of liquid diet nutrition [7].

### 2.2 Outcomes and follow-up

**Fig. 4:**
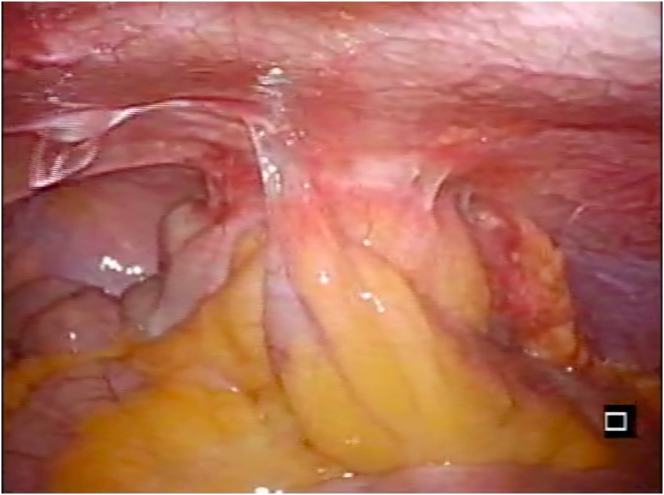
Laparoscopic findings obtained during the definitive surgery. The stenotic area of the ascending colon had adhered to the abdominal wall.

**Fig. 5:**
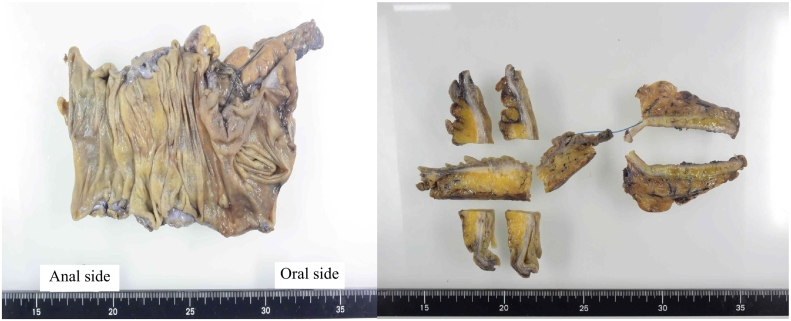
Resected specimen. A thickened and fibrous bowel wall was observed. Bowel inflammation had penetrated into the peri-intestinal adipose tissue, leading to tissue thickening and fibrosis, which had strangulated the gut lumen, resulting in stenosis.

The stenotic region of the ascending colon had adhered to the abdominal wall (Fig. 4), but no necrosis was observed during surgery. A microscopic examination of the excised specimen revealed lots of diverticula surrounding a foreign body granuloma, inflammatory cell infiltration, fibrosis, and hyperemia (Fig. 5). A definitive diagnosis of ascending colon stenosis caused by repeated diverticulitis was made. No postoperative complications were experienced. Normal meals were re-started 5 days after the definitive surgery, and the patient was discharged on the 23rd day of his hospitalization.

## 3 Discussion

Most of the reported cases of LBO caused by diverticulitis were left-sided. In addition to our case, we found 6 other reported cases of right-sided LBO with diverticulitis (Table 1) during a search of PubMed and the Japanese medical journal database (Ichushi) conducted in December 2021 for articles published between 1964 and 2021 using the following search terms: [ascending/right] [colon] [stenosis/obstruction] [diverticulitis]. Thus, right-sided LBO with diverticulitis is a remarkably rare condition. The median age of the patients in the reported cases (including our case) was 53 years, and 5 of the 7 cases involved males. Five patients had stenosis at sites ranging from the cecum to the lower ascending colon, while our patient had stenosis in the upper ascending colon. Surgical resection of the causative lesions was required in all cases. In 3 cases, scheduled surgery was conducted, whereas emergent surgery was performed in one case (the nature of the surgery was unclear in the other cases). Perioperative complications were not reported in detail in all cases.

**Table 1:** Reported cases of right-sided LBO caused by diverticulitis.

Case	Author	Year	Age	Sex	Chief compliant	Location	Surgical procedure	Other clinical findings
1	K. HASE [8]	1987	71	F	Data not available	Data not available	Right hemicolectomy	
2	Y. UCHIDA [9]	2001	35	M	Right abdominal pain	Cecum	Right hemicolectomy	Colonic muco-submucosal elongated polyp
3	M. OKUBO [10]	2006	49	M	Right abdominal pain	Cecum	Ileocecal resection	Lipohyperplasia
4	K. TANIGUCHI [11]	2008	55	M	Chronic abdominal pain	Cecum ~ Ascending colon	Ileocecal resection	
5	K. FURUKAWA [12]	2011	53	M	Right abdominal mass and pain	Cecum ~ Ascending colon	Right hemicolectomy, Partial small bowel resection	Inflammatory fibroid polyp
6	Y. FUKITA [13]	2018	66	F	Right abdominal pain	Cecum	Right hemicolectomy	Chronic diverticulitis
7	Our case	2020	43	M	Right abdominal pain	Ascending colon	Right hemicolectomy	

In addition to our case, 6 other cases were found in PubMed or the Japanese medical journal database (Ichushi). In 3 cases, scheduled surgery was conducted, whereas emergent surgery was performed in one case (the nature of the surgery was unclear in the other cases). Perioperative complications were not reported in detail in all cases.

In the present case, infectious colitis was initially considered as a differential diagnosis due to the acute onset of the patient's symptoms in the absence of previous similar episodes. Some bacteria, such as *Escherichia coli* O156:H7, Salmonella, and Campylobacter, are known to cause inflammation, especially in the ascending colon. *Escherichia coli* O156:H7 infections can cause ascending colon wall thickening of >20 mm on plain CT, but such thickening is generally accompanied by hematochezia and small amounts of ascites [17]. Our patient showed atypical CT findings. In diverticulitis, the structure of the intestinal wall is preserved and diverticula are seen, while cancer commonly causes the collapse of the structure of the intestinal wall [15], [16]. In addition, he did not have any history of diverticulitis or recurrent abdominal pain and showed a slightly elevated CEA level, even though his daily smoking habit could have raised his CEA level [14]. These facts made us to hesitant to rule out advanced cancer [5].

Based on this case, we would like to highlight the importance of “interval surgery”. Our patient was diagnosed correctly and recovered steadily without any complications after definitive surgery because we had decided to perform minimal interventions; i.e., so-called “interval surgery”. In cases of complicated appendicitis, such as those involving perforated appendicitis, we usually choose interval appendectomy [18]. Likewise, choosing conservative treatment rather than immediate definitive surgery, even though surgical interventions are inevitable for LBO, allowed us to scrutinize the disease and diagnose it correctly. Moreover, preoperative nutritional therapy has been reported to reduce surgical complications [7], and subsequent elective surgery was shown to alleviate local inflammation and consequently shorten the duration of hospitalization and reduces recourse utilization [19], [20]. Emergent surgery can cause a variety of complications, which are associated with higher mortality and morbidity [20]. Therefore, performing interval surgery over as long a period as possible can be considered.

## 4 Conclusion

We reported a rare case of right-sided colon diverticulitis with LBO. Clinicians should recognize that diverticulitis is one of the causes of LBO. In addition, interval surgery supposed to be considered to achieve the best outcomes for such patients.

## Sources of funding

None.

## Ethical approval

N/A.

## Consent

Written informed consent was obtained from the patient for publication of this case report and accompanying images. A copy of the written consent is available for review by the Editor-in-Chief of this journal on request.

## Author contributions

Kazuhiro Hiyama performed all clinical interventions and critically revised the final manuscript.

Shogo Yoshida made substantial contributions to conception, design, data acquisition and interpretation of the study and was involved in initial drafting of the manuscript.

Other authors were involved in revision of the final manuscript.

All authors reviewed the final manuscript and give approval of the version to be published and agree to be accountable for all aspects of the work in ensuring that questions related to the accuracy or integrity of any part of the work are appropriately investigated and resolved.

## Research registration

N/A.

## Guarantor

Kazuhiro Hiyama.

## Provenance and peer review

Not commissioned, externally peer-reviewed.

## Declaration of competing interest

The authors declare that they have no conflicts of interest and that there are no relevant financial disclosures to report.
